# Aerobiology Over Antarctica – A New Initiative for Atmospheric Ecology

**DOI:** 10.3389/fmicb.2016.00016

**Published:** 2016-02-16

**Authors:** David A. Pearce, Irina A. Alekhina, Aleks Terauds, Annick Wilmotte, Antonio Quesada, Arwyn Edwards, Aurelien Dommergue, Birgit Sattler, Byron J. Adams, Catarina Magalhães, Wan-Loy Chu, Maggie C. Y. Lau, Craig Cary, David J. Smith, Diana H. Wall, Gabriela Eguren, Gwynneth Matcher, James A. Bradley, Jean-Pierre de Vera, Josef Elster, Kevin A. Hughes, Lewis Cuthbertson, Liane G. Benning, Nina Gunde-Cimerman, Peter Convey, Soon Gyu Hong, Steve B. Pointing, Vivian H. Pellizari, Warwick F. Vincent

**Affiliations:** ^1^Faculty of Health and Life Sciences, Northumbria UniversityNewcastle-upon-Tyne, UK; ^2^British Antarctic SurveyCambridge, UK; ^3^Arctic and Antarctic Research InstituteSaint Petersburg, Russia; ^4^Australian Antarctic DivisionKingston, TAS, Australia; ^5^University of LiegeLiège, Belgium; ^6^Universidad Autónoma de MadridMadrid, Spain; ^7^Aberystwyth UniversityAberystwyth, UK; ^8^Université Grenoble Alpes, GrenobleFrance; ^9^University of InnsbruckInnsbruck, Austria; ^10^Brigham Young UniversityProvo, UT, USA; ^11^Interdisciplinary Centre of Marine and Environmental Research, University of PortoPorto, Portugal; ^12^International Medical UniversityKuala Lumpur, Malaysia; ^13^Department of Geosciences, Princeton UniversityPrinceton, NJ, USA; ^14^University of WaikatoHamilton, New Zealand; ^15^NASA Ames Research CenterMoffett Field, CA, USA; ^16^Colorado State UniversityFort Collins, CO, USA; ^17^Universidad de la RepúblicaMontevideo, Uruguay; ^18^Rhodes UniversityGrahamstown, South Africa; ^19^University of BristolBristol, UK; ^20^German Aerospace CenterCologne, Germany; ^21^University of South BohemiaČeské Budějovice, Czech Republic; ^22^Institute of Botany of the Academy of Science of the Czech RepublicTřeboň, Czech Republic; ^23^Northumbria UniversityNewcastle-upon-Tyne, UK; ^24^Helmholtz Centre Potsdam GFZ, German Research Centre for GeosciencesPotsdam, Germany; ^25^University of LjubljanaLjubljana, Slovenia; ^26^Korea Polar Research InstituteIncheon, South Korea; ^27^Auckland University of TechnologyAuckland, New Zealand; ^28^Universidade de Sao PauloSao Paulo, Brazil; ^29^Laval UniversityQuébec, QC, Canada

**Keywords:** aerobiology, Antarctica, metadata, biodiversity, biogeography

## Abstract

The role of aerial dispersal in shaping patterns of biodiversity remains poorly understood, mainly due to a lack of coordinated efforts in gathering data at appropriate temporal and spatial scales. It has been long known that the rate of dispersal to an ecosystem can significantly influence ecosystem dynamics, and that aerial transport has been identified as an important source of biological input to remote locations. With the considerable effort devoted in recent decades to understanding atmospheric circulation in the south-polar region, a unique opportunity has emerged to investigate the atmospheric ecology of Antarctica, from regional to continental scales. This concept note identifies key questions in Antarctic microbial biogeography and the need for standardized sampling and analysis protocols to address such questions. A consortium of polar aerobiologists is established to bring together researchers with a common interest in the airborne dispersion of microbes and other propagules in the Antarctic, with opportunities for comparative studies in the Arctic.

## Introduction

**FIGURE 1:**
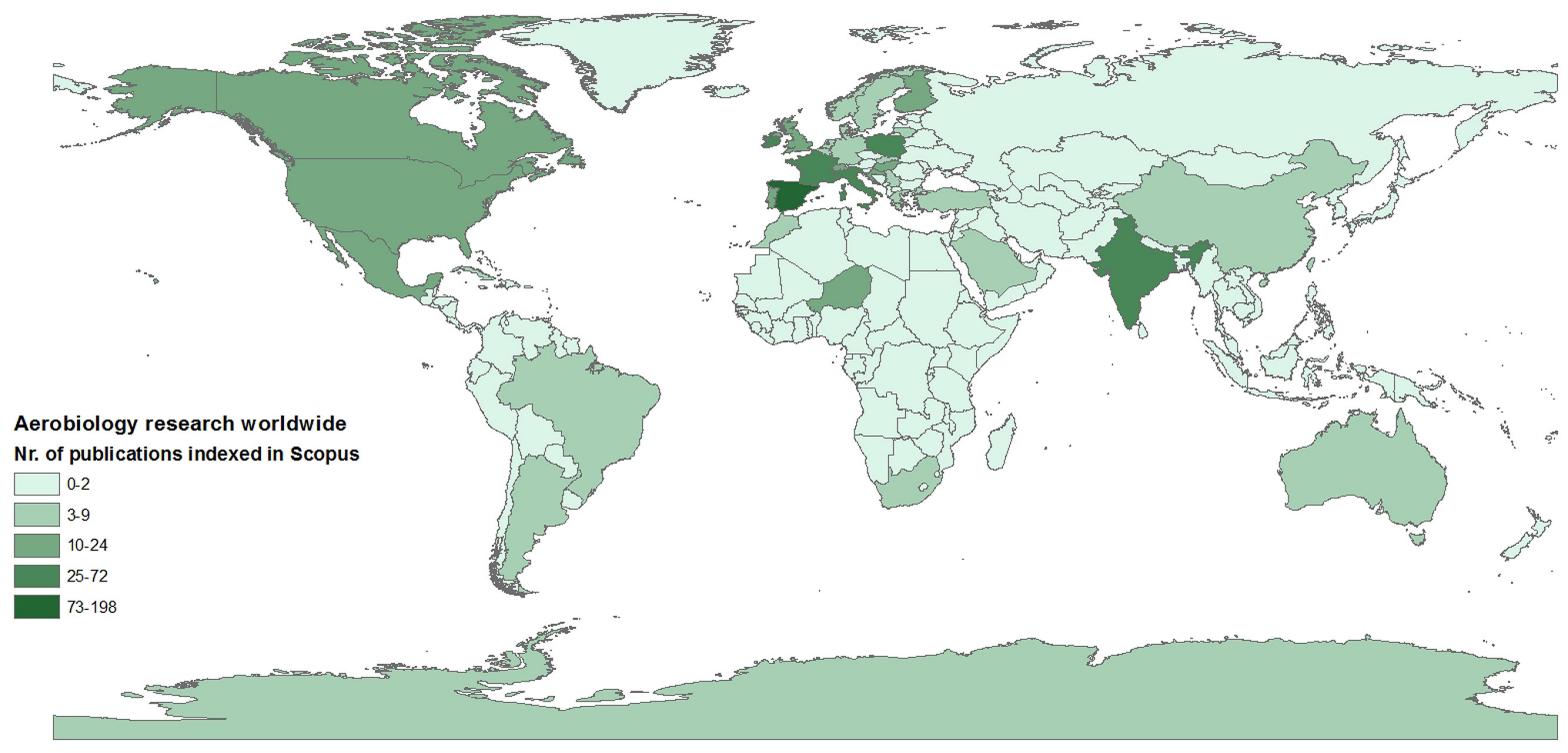
**Distribution of aerobiological studies worldwide to date.** The map shows the number of aerobiology studies published in English (and indexed in Scopus), as a measure of the uneven and scattered distribution of aerobiological studies worldwide.

Aerial dispersal plays an essential role in shaping patterns of biodiversity (e.g., Womack et al., 2010). However, the ability of atmospheric ecology to help understand large scale patterns of biodiversity remains limited, mainly due to a lack of coordinated efforts in gathering data at appropriate temporal and spatial scales (**Figure 1**). It has been known for some time that the rate of dispersal to an ecosystem can significantly influence ecosystem dynamics; indeed, aerial transport has been identified as an important source of biological input to remote locations (e.g., Pearce et al., 2010). With the considerable effort devoted in recent decades to understanding Antarctic atmospheric dynamics, we believe a unique opportunity has emerged to investigate atmospheric ecology from regional to continental scales.

Despite the acknowledged importance of airborne microorganisms (including microscopic spores and other propagules) (Fierer, 2008), most aerobiological studies have consistently failed to consider the stability and viability of wind-borne microorganisms in the aerial environment. Whilst it is assumed that potential colonists arrive continually from the atmosphere, for example, linked to precipitation and wind-blown debris, the often extreme and selective nature of the atmospheric environment is likely to limit the viability of the material transported to an unknown extent. With evolution, extinction, and colonization driving microbial biodiversity patterns, aerial dispersal becomes intimately linked with eco-evolutionary dynamics across terrestrial, freshwater, and marine environments. Consequently, knowledge of the rates of airborne input, survival of the imposed stresses of the transfer process, and viability on arrival, is essential for understanding ecosystem stability and resilience.

**FIGURE 2:**
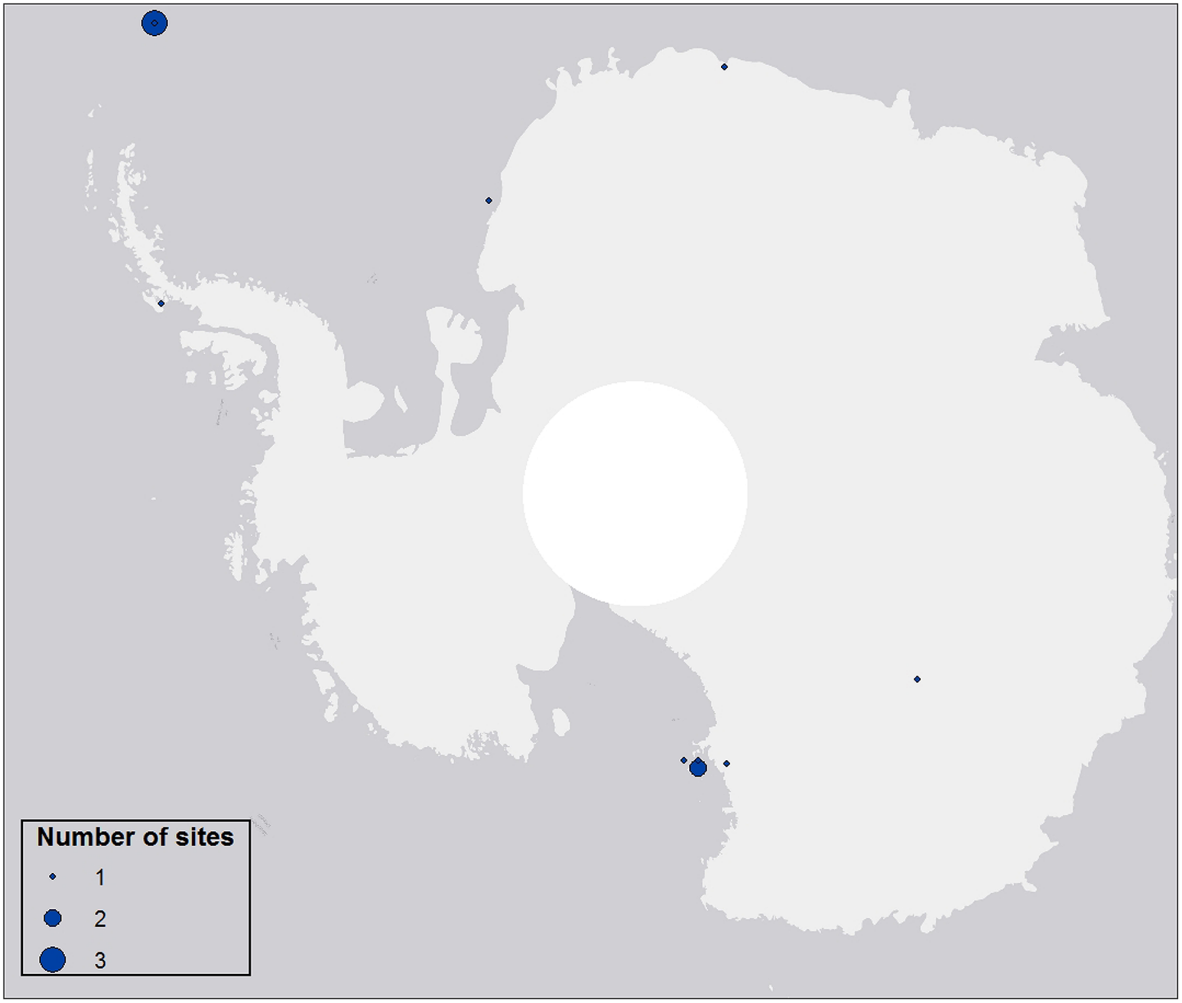
**Distribution of aerobiological studies over Antarctica.** Data extracted from studies indexed in ISI Web of Science and those available to the authors but not indexed, published between 1994 and 2014. A total of 12 studies were included. No studies prior 1994 were available in ISI Web of Science. Circle diameter indicates the number of sites included per study.

Aerial biodiversity studies carried out to date have generally been based on single-site investigations over limited time periods, providing ‘snapshot’ information on the abundance, distribution and diversity of microorganisms found in specific aerial environments (e.g., Pearce et al., 2010; **Figure 2**). Although these have confirmed the magnitude of aerial dispersal, they have failed to address its influence on ecosystem stability and resilience, only providing qualitative data in this regard.

A changing climate leads to changes in the frequency, intensity, spatial extent, duration, and timing of extreme weather and climate events (IPCC, 2012), so understanding the direct link between weather conditions and biological dispersal is essential to determine the rate of climate-driven ecological change worldwide. Here, we present a suggested minimal methodology intended to gather wide ranging metadata relevant to aerial ecology at representative temporal and spatial scales. The methodological approach discussed here, and agreed by the pan-Antarctic initiative ‘Aerobiology over Antarctica’, provides a series of sample handling guidelines and metadata characteristics required to ensure pan-Antarctic and worldwide sampling consistency, and represents the first-ever coordinated effort to provide a dynamic global map of aerobiological transport.

### The ‘Aerobiology Over Antarctica’ Consortium

With recent agreement to co-ordinate weather and climate monitoring at the XIth Scientific Committee on Antarctic Research (SCAR) symposium – Life in Antarctica: Boundaries and Gradients in a Changing Environment, Barcelona, 15–18th, July 2013, the necessary foundation exists to enable establishment of a pan-Antarctic sampling initiative. For the first time, this initiative encompasses a co-ordinated program to produce (i) a global dataset on aerobiological diversity and (ii) contextualized environmental data aimed at clarifying the relationship between aerial biodiversity and terrestrial ecosystem stability. At the XXXIIIth SCAR Open Science Conference, Auckland, New Zealand, 23rd August – 3rd September 2014, a workshop was held to discuss the structure, sampling, and environmental data recording methodologies, and common approaches to data analyses that would be fundamental to the success of such a program, and would render it technically feasible while also minimizing costs. Aerobiological samplers are relatively light and easy to install, monitor and use, with minimal power requirements. Furthermore, it is only relatively recently that the logistic potential has existed to launch a co-ordinated continental (Antarctic) or even global field sampling campaign. The analytical technology required for such an undertaking has only become widely available with the advent of high-throughput DNA sequencing. This has allowed a departure away from reliance solely on the more traditional culture-based microbiological approaches, permitting a systematic analysis of the diversity of marker gene sequences and generating data that are amenable to rigorous statistical analysis.

Initial discussions on program development have involved participants representing 27 institutions from 19 countries. The key challenge in this type of study, as for many studies in microbial ecology, is that the abundance and composition of airborne communities is variable across time and space. This means that a large area (global or pan-continental) aerobiological sampling initiative could be compromised by the specific methods selected and the techniques used in different regions. To overcome such challenges, we propose the use of standardized minimal air collection and sample processing methodologies and statistical analyses, in order to identify and detect patterns in aerobiological datasets obtained from a wide variety of sources and approaches.

### The Atmosphere as Habitat for Microorganisms

Viable atmospheric biota are often assumed to be dormant and in a cryptobiotic state, with active metabolism impossible in these harsh dry, low nutrient, high irradiance growth conditions. Although a number of studies challenge this paradigm (e.g., Sattler et al., 2001), atmospheric diversity and ecology, and the critical microbial biomass required to colonize a particular environment and effectively influence its ecological dynamics, remain unexplored. Antarctic studies to date seem to suggest a strong relationship between aerial propagules and terrestrial flora (e.g., Hughes et al., 2004), highlighting the need to understand the nature and direction of these interactions.

Airborne microorganisms may play an important role in the global climate system by absorbing or reflecting incoming sunlight, acting as cloud condensation nuclei or serving as ice nucleating particles (see e.g., Möhler et al., 2007). Their metabolic reactions can alter the atmosphere’s chemical composition, including the production of carboxylic acids from common atmospheric compounds (Amato et al., 2007). Using incubation of cloud water, a recent study highlighted the activity of microorganisms as an alternative route in photochemistry and showed that they significantly alter OH radical production via H_2_O_2_ degradation (Vaïtilingom et al., 2013). In addition, once deposited on snow, microbes may participate in and alter other biogeochemical cycles (e.g., Maccario et al., 2014).

### Biogeography of Microorganisms

While progress has been made in microbial biogeography with respect to categorizing the observed microbial distribution in space and time (Fierer and Jackson, 2006; Martiny et al., 2006; O’Malley, 2007; King et al., 2010; Wilkinson et al., 2012; Lutz et al., 2015, under review), especially for single species, we are still far from a complete understanding of the factors that control the process. Yet, invasions by non-indigenous species have been identified amongst the greatest threats to global biodiversity (Litchman, 2010) particularly in response to disturbance and this, in turn, can affect ecosystem structure and function. There is also the issue of airborne human disease outbreaks. One of the mechanisms to explain microbial biogeographic patterns is dispersal. However, there are limited empirical observations to support the role and significance of air dispersal that has been hypothesized in microbial biogeography. Aerobiology and concurrent research on local features *en route* of the air mass transport, is therefore important to provide evidence of connections between the airborne microbial assemblages and biota in surface habitats. As a consequence, there are still major gaps in our understanding of airborne microbial diversity and distribution, and the potential influence of airborne strains on the underlying terrestrial environment (Womack et al., 2010).

### Using Antarctica to Investigate Global Microbial Dispersal

Antarctica is the most remote continent on Earth. Its isolation from the rest of the world through the Southern Ocean’s Antarctic circumpolar current and the atmospheric circumpolar vortex and ‘west wind drift’ makes it particularly well suited for studies involving the aerial transport and survival of microorganisms and other transported biota (Siegert et al., 2008). Previous studies (see e.g., Vincent, 2000) have discussed the frequent transfer of biological material to Antarctica by atmospheric processes. However, little is known about the contribution of bioaerosol transport to the microbial ecology of isolated systems on the Antarctic continent (Bottos et al., 2014). Data on long-distance dispersal of airborne organisms by trade winds are limited for microbes dispersed into the Antarctic environment (Hughes et al., 2004), as well as data on their viability, duration of suspension and gravitational settlement. In addition, the origin and maintenance of endemic populations in isolated regions implicitly must be indicative of a (low) rate of airborne exogenous inputs (i.e., a lack of genetic homogenization), although this has proven hard to confirm and, rather, distinct bio-aerosol communities are often reported (e.g., Bottos et al., 2014). On the other hand, the high percentage of biological provinces endemic to specific Antarctic areas may be an artifact caused by the lack of continental-wide biodiversity surveys. Ultimately, its level of isolation, combined with an extreme environment able to challenge the viability of long-range colonists, and the presence of widely distributed groups (such as cyanobacteria, diatoms, ciliates, rotifers, crustaceans in freshwater systems, and terrestrial invertebrates, bryophytes and lichens), many of which are typified by cryptobiotic life stages and/or resistant dispersing propagules, makes the Antarctic an ideal platform for this type of study. Antarctic environments are also among the least human-modified terrestrial ecosystems on earth, enabling accurate interpretation of patterns of genetic diversification or dispersal. These relatively simple terrestrial ecosystems allow ecological communities to be surveyed in unprecedented detail, to an extent not feasible in more species-rich ecosystems. Snow and ice have largely low levels of microbial life compared to marine or terrestrial environments. This makes interpretation of data collected on Antarctic ice-free ‘islands’ more straightforward, i.e., the background contamination between propagule source and those collected/detected at the destination is greatly reduced compared to other parts of the planet.

## Methods

A balance needs to be struck between the main aim of the consortium – to encourage the collecting of metadata of as wide a variety of types as possible and also a practical suggestion for those who seek guidance on methodology. A suggested method is summarized in **Table 1**, but it should be noted that this is a suggestion and not a recommendation or consensus.

**Table 1:** Summary of the proposed method and contextual data.

Method	
Sampling	Active accumulation onto dry 0.2 μm 47 mm diameter sterile polycarbonate filters supported by a variety of different sampling methods to enhance the quality of the data.
Sampling platforms	Aim for 3 m above ground level to minimize local effects, whilst still being supported by a variety of different sample heights to enhance the quality of the data.
Scale of sampling	Target all microorganisms and biological material containing DNA. A minimum of three replicates per site and as wide coverage as is practical.
Duration of sampling	Sample a minimum of 24 h assay for biomass and extend as long as practical.
Sample integrity	Use best practice feasible for the field location in question. The essential component here is an accurate and detailed description of the methodology employed.
Method of analysis	Microscopy, culture and DNA extraction and analysis using high throughput sequencing. Here, for instance, we suggest the V3–V4 hypervariable region (Caporaso et al., 2012) for the simultaneous detection of bacteria and archaea. We also suggest including shotgun metagenomic analysis which will cover all groups and functions including 18S and virus markers. Some form of biomass quantification is desirable.
**Contextual data**	
Meteorological data	By collaborating with a multi-national continent-wide observing system ensure that sampling sites are congruent with environmental monitoring stations. This will provide a suite of parameters that can be used to clarify the links between airborne microbes and the associated physical environment.
Modeling	Use tested and contemporary models to clarify the relationship between airborne microbe biodiversity and associated environmental parameters.
Reproducibility	Repeat sampling at intervals throughout the year and in multiple years as logistic opportunity permits.
Data management	Adopt mARS and utilize specialist public culture collection repositories.


### Sampling

The results generated by aerobiological sampling depend heavily upon the sampling method used. This can be either passive, allowing particles to collect through natural processes such as air movement or gravity, or active, where large volumes of air are passed over or through a means of entrapment (reviewed by Griffin et al., 2011). Methods range through simple drop plates (which can be augmented by different selective media), suction onto dry or gelatine filters (either via commercial aerobiological sampling equipment or simple pump systems), to the many different impactor approaches (i.e., solid and liquid). Whilst one outcome of the Auckland workshop was a recommendation for active accumulation onto a 0.2 μm 47 mm diameter polycarbonate filter, it is clear that a variety of different sample methods would also be useful to assess sampling bias. The ideal approach depends on whether the information needed is qualitative or quantitative, highly specific or of a general nature, highly localized or over a broader landscape. It also depends on funding in the researcher’s country, logistic field opportunities, and on ground support. The combined strengths of selective culture, multiplexed molecular methods, high-throughput sequencing and new instrumentation are improving our ability to simultaneously detect a wide variety of organisms against a complex and variable natural background. Despite clear differences on the merits and limitations of different methods, there is no clear consensus on an ideal approach. The more traditional methods, including culturing on selective media, continue to have utility as they demonstrate viability of those cells amenable to culture, although as is well known not all viable cells will grow. As technology advances in microbial ecology, so do the approaches available, for example the application of real-time quantitative PCR (e.g., Smith et al., 2012) and metagenomic analyses (e.g., Smith et al., 2013).

### Sampling Platforms

There are a very wide variety of possible sampling platforms, from ground level to high altitude, and from the individual scientist with a single plate to an aircraft or weather balloon custom-fitted to collect air samples. Sampler positioning will influence the material that is collected, as will the existence of local obstructions (i.e., topography) which might induce turbulence. For the project wider data collection, variety is the key. Different projects will use different methodologies, and it is the diversity of these different sampling platforms which will add strength to the data collected. It is anticipated, though, that most might be sampled close to weather stations, and below c. 5 m in altitude, for practical reasons. Where possible, care should be taken to try and account for transfer of the biota between the near-surface atmosphere and the boundary layer just above the ground surface. For instance, rather than sampling at a single height, important relevant information would be generated by deploying paired samplers at approximately 3 m (for capturing long-range dispersed microbiota) and at c. 0.3 m to detect those near to the event of landing.

### Scale of Sampling

It is well documented that air samples collected from different locations may differ with respect to the relative abundances of specific bacterial and fungal groups (e.g., Marshall, 1996). The information obtained through this initiative at the Antarctic continental level, including inflow and outflow, will provide a robust foundation for eventual scaling up to the global level. Sampling will inevitably include air masses that move into, around, and away from the continent; however, individual studies might range from a single sample or small numbers of samples taken every few meters, to sampling locations separated by 100s of kilometers, depending on the nature of the particular project. Coverage will be the key here, and analyses at different spatial scales will enhance the quality of the data. The advantage of using DNA as a target molecule for biodiversity studies is that it does not exclude different target groups: viruses, prokaryotes and eukaryotes.

### Duration of Sampling

The time spent sampling is important for aerobiology, as propagules can be assayed per liter of air. Sample times may range between a few seconds and a few years. Longer sampling times should yield higher numbers of propagules. Fierer (2008) demonstrated short-term temporal variability in airborne bacterial and fungal populations. Results suggest that outdoor air could harbor similar types of bacteria, regardless of location, and that the short-term temporal variability in airborne bacterial assemblages can be very large. For particularly low biomass systems such as the Antarctic, it is expected that large volumes might be needed as propagule density is typically several orders of magnitude lower than that typical over lower latitude continents (Burrows et al., 2009). This requires a trade-off between sampling periods short enough to avoid desiccation or damage to samples against long enough to sample sufficient biomass to give meaningful data. To this end, Durand et al. (2002) investigated the effect of sample time on the culturability of airborne fungi and bacteria sampled by filtration, reporting no loss in viability. There are already studies of this type, and it is anticipated that a variety of approaches will enhance the quality of the data.

### Sample Integrity

Aerobiological studies have sometimes been hampered at the publication stage by sample integrity. In an ideal world, the aspiration would be to use completely sterile sampling equipment, avoid any human contact, and process all material in a dedicated and certified clean laboratory. However, this is not always practical, especially under Antarctic field conditions. Attempts can be made to minimize contamination of sample material, such as the use of sterile materials, stringent procedural negative and positive controls, the use of barrier type personal protective equipment, and by returning sealed samples under sterile conditions for processing in more controlled laboratories in researchers’ home countries. However, further analyses of the data obtained might increase understanding of the nature of in-process contamination risks. Indeed, such contaminants (i.e., microorganisms brought in as a consequence of researcher activity) are part of the contemporary Antarctic environment and so may, themselves, be considered a valid research target and an important part of the analyses carried out (Pearce et al., 2010; Cowan et al., 2011; Hughes et al., 2011).

### Method of Analysis

All methods in microbiology, without exception, are subject to bias and limitations, and this means that a polyphasic approach is often the only way to ensure the reliability of results. The most frequently used aerobiological techniques are culture, microscopy, and DNA extraction followed by high-throughput sequencing. For the studies we propose, a polyphasic approach is indeed optimal; however, some co-ordination would be helpful in the final analysis, such as the selection of the same DNA extraction methodologies and homologous gene regions for high-throughput sequencing.

## Contextual Data

### Meteorological Data

In order to make sense of the aerobiological diversity, it is important to collect environmental context data. In combination with backtrack analyses, researchers also need to consider the conditions the air mass has or will experience *en route* between two regions, not just those at the ‘landing site’, as these will determine survival during transfer. Collaboration with current platforms, such as the MCM TON (McMurdo Terrestrial Observation Network – these networks are being designed to monitor key physical and biological processes associated with changing ecosystems across regional to continental spatial scales by facilitating coordination and comparability of measurements) and ANTOS (SCAR Antarctic Nearshore and Terrestrial Observing System) initiatives, would help generate a standard suite of environmental parameters.

Relevant parameters include wind speed (instantaneous and over time), direction, fetch, humidity, precipitation, barometric pressure (Woo et al., 2013), light and ultra-violet intensity, storm proximity (Marshall, 1996), location (for proximity to potential terrestrial, and marine inputs), temperature, composition (e.g., moisture, salt content, dust inputs), and chemistry (e.g., ozone, ice nucleating agents).

### Modeling

Different numerical models have been used in aerobiology over a range of applications, including pollen dispersal (and allergy susceptibility), species invasions, spread of diseases and air pollution (see e.g., Garcia-Mozo et al., 2009). These models represent useful tools to test current ecological hypothesis. For example, data from the Antarctic Mesoscale Prediction System (AMPS) and the application of NOAA HySPLIT system could be used to create back trajectories over the Antarctic continent, indicating the sources of particular air masses and, potentially, their contained biota. The co-ordinated sampling approach outlined here would provide observational data for use in modeling studies (including community compositions and species distributions), particularly if combined with meteorological data (Westbrook, 2010). Fitted models, including Structural Equation Models (SEM), Generalized Linear Mixed Models (GLMMs), and Simultaneous Autoregressive Models (SARs) can in addition take into account spatial autocorrelation.

### Reproducibility

Most studies completed to date have inevitably involved one-off or opportunistic sampling. The data generated through this initiative, and classified in the form of metadata, might allow the reproducibility of sampling to be assessed. It is essential to know whether the observations are random, or whether patterns are apparent in the observations that can be attributed to specific environmental characteristics. Previous researchers (e.g., Smith et al., 2012, 2013) have addressed seasonal variability in airborne bacterial communities. They examined seasonal shifts in microbial abundance and viability, and independently observed seasonality corresponding to highest concentrations of bioaerosols.

### Data Management

The datasets likely to be generated by this initiative are large but not necessarily complex. A number of data management initiatives are already underway though SCAR, and would be appropriate to utilize here. For example, the Microbial Antarctic Resource System (mARS) for sequence data (using MIMARK environmental data format guidelines), the Polar and Alpine Microbial Collection (KOPRI, Korea), the collection of polar cyanobacteria BCCM/ULC (Liege, Belgium), the DNA repository for long term DNA storage (University of Waikato, New Zealand), the SCAR Antarctic Terrestrial Biodiversity Database (Australian Antarctic Data Centre), and the Antarctic Plant Database (held at the British Antarctic Survey).

### Next Steps

To get involved, register your project with the consortium. We will develop and host a metadata repository to identify ongoing and prospective studies, that can be used to suggest links and collaborations that can lead to enhanced datasets. Registrants will have the opportunity to become contributors to a coordination workshop to analyze and develop the next stage of implementation of the project ‘Gathering large scale spatial and temporal airborne microbial samples to understand the role of airborne input to continental Antarctic ecosystem function, its resilience and stability’.

## Author Contributions

All authors listed, have made substantial, direct and intellectual contribution to the work both through active participation in the original workshop or in subsequent development discussions, and approved it for publication.

## Conflict of Interest Statement

The authors declare that the research was conducted in the absence of any commercial or financial relationships that could be construed as a potential conflict of interest.
